# Heat stress response and transposon control in plant shoot stem cells

**DOI:** 10.1093/plphys/kiaf110

**Published:** 2025-03-28

**Authors:** Vu Hoang Nguyen, Ortrun Mittelsten Scheid, Ruben Gutzat

**Affiliations:** Gregor Mendel Institute of Molecular Plant Biology, Austrian Academy of Sciences, Vienna Biocenter (VBC), Vienna 1030, Austria; Gregor Mendel Institute of Molecular Plant Biology, Austrian Academy of Sciences, Vienna Biocenter (VBC), Vienna 1030, Austria; Gregor Mendel Institute of Molecular Plant Biology, Austrian Academy of Sciences, Vienna Biocenter (VBC), Vienna 1030, Austria

## Abstract

Plants have an impressive repertoire to react to stress conditions that limit regular growth. Elevated temperatures beyond the optimal range cause rapid and specific transcriptional responses, resulting in developmental alterations and plasticity. Heat stress also causes chromatin decondensation and activation of some transposable elements (TEs), endangering genomic integrity. This is especially risky for stem cells in the shoot apical meristem (SAM) that potentially contribute to the next generation. We examined how the heat stress response in SAM stem cells of Arabidopsis (*Arabidopsis thaliana*) is different from that in other tissues and whether the elements of epigenetic TE control active in the meristem are involved in specific heat protection of stem cells. Using fluorescence-activated nuclear sorting to isolate and characterize SAM stem cells after exposure to conditions that activate a heat-responsive TE, we found that SAM stem cells maintain their developmental program and suppress the heat-response pathways dominating in surrounding somatic cells. Furthermore, mutants defective in DNA methylation recovered less efficiently from heat stress and persistently activated heat response factors and heat-responsive TEs. Heat stress also induced epimutations at the level of DNA methylation, especially in the CHG sequence context. Regions with modified DNA methylation patterns remained altered for at least 3 wk beyond the stress. These findings urge for disentangling cell type-specific responses under different stress types, especially for stem cells as bridges to the next generation.

## Teaser

In heat-stressed Arabidopsis, shoot meristem stem cells preserve their developmental programs, while surrounding somatic cells exhibit a strong heat response.

## Introduction

While plants can compensate for low temperatures to some degree by arresting growth and development, too high temperatures can cause irreversible damage to proteins, membranes, cells, and tissues. Numerous studies have described sophisticated pathways of heat signaling and transcriptional responses that help plants limit the damage and allow recovery once the heat stress has passed (for review, e.g. Zhao et al. 2020; Zhou et al. 2022; Kan et al. 2023). At the same time, heat stress can weaken epigenetic regulation by modifying DNA methylation, histone modifications, or large-scale chromatin organization (e.g. Pecinka et al. 2010; Liu et al. 2022; Perrella et al. 2022; Kappel et al. 2023; Torres et al. 2023). This opens a window of opportunity for activation of otherwise epigenetically controlled transposable elements (TEs). One example is the retrotransposon *ONSEN* of Arabidopsis (*Arabidopsis thaliana*) which becomes transcribed at high temperatures (Tittel-Elmer et al. 2010). *ONSEN* has copied heat-responsive elements from plant genes into its regulatory regions and forms extrachromosomal DNA upon heat stress (Cavrak et al. 2014). In mutants lacking components of epigenetic control mechanisms, these extra copies can integrate into new genomic positions in the progeny of heat-stressed parents (Ito et al. 2011; Pietzenuk et al. 2016; Hayashi et al. 2020; Niu et al. 2022; Nozawa et al. 2022). Studies with somatic tissues (Sun et al. 2020; Guo et al. 2021) and gametophytes (Slotkin et al. 2009; Olmedo-Monfil et al. 2010) showed that transposon control can differ between somatic and germline cells. The patterns of new *ONSEN* insertions differed between seeds obtained from individual flowers but were similar within the progeny from the same flower (Ito et al. 2011), indicating that transposition happened in somatic tissue before gamete specification. When and where remains unknown and is difficult to analyze, as the lineage from parental plant cells to progeny gametes is unclear and debated (Lanfear 2018). However, this lineage must include stem cells in the shoot apical meristem (SAM) that develop inflorescence meristems and finally form gametes. There is evidence that plants have evolved factors that protect stem cells in meristems against heat stress (Olas et al. 2021). We have previously shown that SAM stem cells resemble germline cells in some respect and are hubs for TE activity (Nguyen and Gutzat 2022; Bradamante et al. 2024). We have now isolated the nuclei of SAM stem cells from plants grown under regular or elevated temperatures and performed transcriptome and methylome analyses in comparison with somatic cells, after heat stress, and after a recovery period. Using *ONSEN* as an indicator for heat stress-induced transposon activation, we asked for specific changes in gene expression, transposon transcripts across all annotated TE element families, and changes in DNA methylation at genes and transposons. To consider the role of 2 main pathways contributing to the epigenetic control of TEs, we included 2 corresponding mutants. (i) *poliv*, lacking the plant-specific RNA polymerase POLIV that produces short transcripts from methylated regions (Zhai et al. 2015). These transcripts are processed by RNA-dependent RNA polymerase 2 (RDR2) and DICER 3 (DCL3) into 24 nt-long siRNAs that are loaded onto nuclear ARGONAUTE (AGO) proteins. Together with the AGOs, the siRNAs guide the RNA-induced silencing complex (RISC), which includes methyltransferases DOMAIN REARRANGED METHYLTRANSFERASE 1/2 (DRM1/2), to complementary transcripts of another plant-specific RNA polymerase, POLV. DRM1/2 methylates DNA at CHH sites (H = A,C,T), mainly at shorter TEs located on chromosome arms (Wendte and Pikaard 2017). (ii) *ddm1* lacks the chromatin remodeler DECREASE IN METHYLATION 1 (DDM1) and has reduced DNA methylation at cytosines in all sequence contexts (Zemach et al. 2013), likely by the failure to install the heterochromatin-associated histone variant H2A.W (Osakabe et al. 2021) or by reducing chromatin access to DNA methyltransferases METHYLTRANSFERASE 1 (MET1) and CHROMOMETHYLASE 2/3 (CMT2/3). Different transposons were shown to be transcribed and reinserted in *ddm1* mutants (Vongs et al. 1993; Hirochika et al. 2000; Kato et al. 2004; Higo et al. 2012). DDM1 has a more prominent role in DNA methylation on long pericentromeric TEs (Zemach et al. 2013).

Our results uncover tissue- and genotype-specific heat stress responses. We find that stem cells are exempted from transcriptional changes that prevail in whole seedlings. Furthermore, we show that both mutants affected in TE silencing are hypersensitive to heat treatment. This suggests that POLIV and DDM1 are critical in suppressing the heat stress response, including heat response factors and heat-induced transposons during recovery. We also detect heat-induced DNA methylation changes in stem cells, especially at CHG sites, in accordance with their dynamic methylation pattern in these cells during development (Gutzat et al. 2020). The changes can persist for weeks after the end of the heat stress, providing a window of opportunity for lasting modifications of epigenetic patterns.

## Results

### Choice of heat stress conditions that activate *ONSEN* and affect methylation mutants

**Figure 1.**
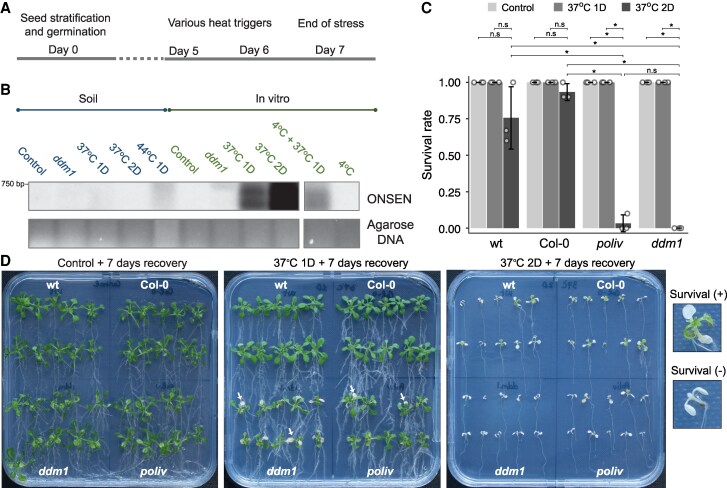
Applied heat stress conditions activate *ONSEN* and affect methylation mutants. **A)** Experimental scheme for testing different heat stress conditions. **B)** Quantification of *ONSEN* extrachromosomal DNA by Southern blot hybridization. Culture medium, temperature, and duration of heat stress are indicated per lane. All samples are from wt (wild-type = Col-0 with stem cell reporter *pCLV3::H2BmCherry*) plants except *ddm1*. Blot and gel section is shown in Supplementary Fig. S2. **C)** Survival rates of Col-0, wt, *poliv*, and *ddm1* plants after 7 d of recovery at 21 °C, with 30 plants each per genotype and 3 biological replicates. Statistical analysis was performed using a two-sided *t*-test. Bars represent standard deviation, n.s indicates *P* > 0.05, * indicates *P* < 0.05. **D)** Images of plants 7 d after heat exposure; right: scoring criteria.

First, we established sublethal heat stress conditions that induce maximal TE activation. As an indicator of response, we measured the degree of *ONSEN* transcriptional activation across different temperature regimes and culture conditions. Heat-activated *ONSEN* forms extrachromosomal DNA from retrotranscribed RNA, which can be quantified by Southern blot analysis (Ito et al. 2011; Cavrak et al. 2014). Plants were cultured on soil or in vitro and exposed to varying temperature regimes (Fig. 1, A and B, Supplementary Figs. S1 and S2). In vitro grown plants contained much more *ONSEN* extrachromosomal DNA than soil-grown plants (Fig. 1B). This discrepancy could be attributed to increased humidity and reduced transpiration cooling within the culture vessels of in vitro grown plants. We also tested a preceding cold treatment, according to previous studies (Ito et al. 2011). However, under our growth conditions, cold treatment (4 °C) neither induced extrachromosomal *ONSEN* on its own nor enhanced *ONSEN* response to subsequent heat treatment (Fig. 1B). The maximum *ONSEN* abundance was observed after heat stress at 37 °C for 2 d (Fig. 1B). We then compared the survival rate as an indicator for the degree of recovery from the preceding heat stress. The stem cell reporter *pCLV3::H2BmCherry* is necessary for the FANS procedure (Gutzat et al. 2020) and was also introgressed into the silencing mutants. All plant material used subsequently is therefore transgenic for the reporter. To increase the readability of text and figures, we refer to the reporter in the Col-0 background as wt. As expected, the nontransgenic Col-0, as well as this line (wt), survived the harsh treatment well (Fig. 1C). Unexpectedly, however, 2 d at 37 °C was lethal for seedlings of the DNA methylation mutants *poliv* and *ddm1* (Fig. 1, C and D). Both mutants showed bleached cotyledons and necrotic spots on leaves and cotyledons after 1 d at 37 °C but survived the recovery period (Fig. 1D). To allow the inclusion of the mutants in the experiments, we chose in vitro growth of the plants and exposure for 1 d at 37 °C, providing strong *ONSEN* induction and survival of all genotypes.

### Gene expression analysis documents transcriptome changes induced by heat stress

We aimed to understand (i) whether gene expression under heat stress differs between stem cells and somatic tissue; (ii) if and how these changes are influenced by DNA methylation; and (iii) whether changes in gene expression or DNA methylation induced by heat stress persist in stem cells, with the potential to become heritable.

To address these questions, we performed mock treatments (C for “Control”) and transiently elevated temperature (H for “Heat”) treatment in wt, *poliv*, and *ddm1* plants, all containing the reporter labeling the nuclei of SAM stem cells. We collected above-ground seedling material (T for “Tissue”) and stem cell nuclei (S for “Stem cells”) using FANS right after the 1-d-long heat stress applied 7 d after germination (“7”) (Supplementary Fig. S1). For wt, we also collected nonstem cell nuclei (N for “Non stem”), representing nuclei of all cells from above-ground tissue. Remaining, nonprocessed seedlings were transferred to soil and grown for an additional 21 d when they started to flower (“28”, 28 d after germination). At this time point, we collected hand-dissected apices with inflorescence meristems (I for “Inflorescence”) and again isolated stem cell nuclei by FANS.

Collecting stem cell nuclei after heat stress was challenging, as they were more sensitive to the extraction procedure than control samples, and heat-stressed tissues displayed more autofluorescence. This resulted in a low yield of intact nuclei in general, including stem cell nuclei. However, we could obtain genome-wide representative transcripts from only 50 nuclei in combination with smart-seq3 single-cell sequencing (Bradamante et al. 2024); therefore, we used bulks of 100 nuclei as samples for FANS-ed material with this approach for all mRNA-seq samples (Supplementary Fig. S3).

**Figure 2.**
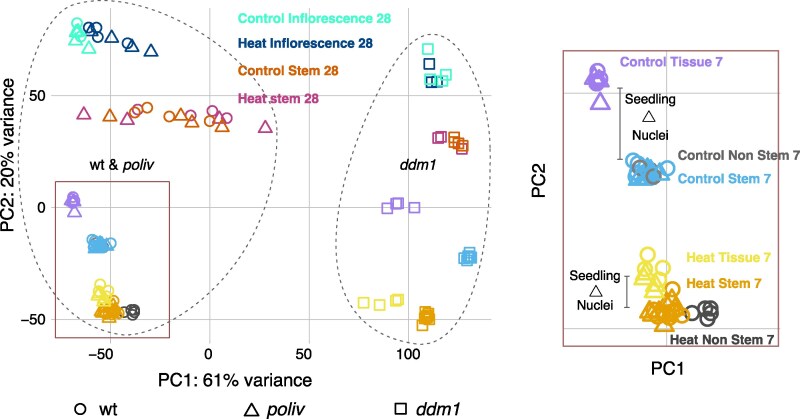
PCA of mRNA data documents gene expression changes induced by heat. wt (wild-type), *poliv*, *ddm1* depict genotypes; Control: grown at regular temperature; Heat: grown at elevated temperature; Inflorescence: whole tissue nuclei (mainly nonstem cells); Stem: sorted stem cell nuclei; Nonstem: sorted nuclei not expressing the stem cell marker; sampled after 7 or 28 d. A magnified view of the boxed area of the PCA plot is presented on the right.

Principal component analysis (PCA) of the mRNA data showed a clear grouping of the samples (Fig. 2, Supplementary Fig. S4). PC1 separated mostly *ddm1* from wt and *poliv* samples, demonstrating the general deregulation of gene expression in this mutant. wt and *poliv* samples always clustered together. PC2 primarily separated tissue types and stress conditions.

Notably, at 7 d after germination, the control samples demonstrated a distinct separation between seedling tissue and nuclei (stem cell nuclei and nonstem cell nuclei), and stem cell nuclei clustered together with nonstem cell nuclei (Fig. 2, inset panel). Upon heat stress, the separation between seedling tissue and nuclei became less pronounced, but stem cell samples were now clearly separated from nonstem cell samples (Fig. 2). This suggests that the expression of a set of genes is altered by high temperature in all tissues, yet with specific differences evident in stem cells.

At the recovery stage, 21 d after heat treatment, heat-treated and control stem cell nuclei overlapped (Fig. 2), implying that most genes had reverted to similar expression states as before the stress.

For all genotypes, there was a prominent difference in gene expression between stem cell nuclei and the surrounding tissue (Fig. 2, “Inflorescence”). Interestingly, a slight separation persisted 21 d after heat treatment for the inflorescence meristems of wt and *poliv* (Fig. 2), which could indicate some long-lasting transcriptome changes. However, we could not observe this in the *ddm1* samples.

Taken together, the results show that the impact of severe heat stress on gene expression can reduce tissue-specific differences but cannot override the substantial *ddm1*-specific differences, connected with reduced DNA methylation in all sequence contexts and severe chromatin decompaction already before heat stress (Probst et al. 2003). Furthermore, the lack of asymmetric DNA methylation in heterochromatin, characteristic of *poliv*, does not substantially influence the global transcriptional heat response. Importantly, the data indicated differences between stem cells and somatic cells immediately after heat stress and between wt, *poliv*, and *ddm1*, and we expected them to be informative in understanding the mutants’ increased heat sensitivity and the dependence of heat-specific responses on DNA methylation.

### Heat response differs between stem cells and somatic cells

**Figure 3.**
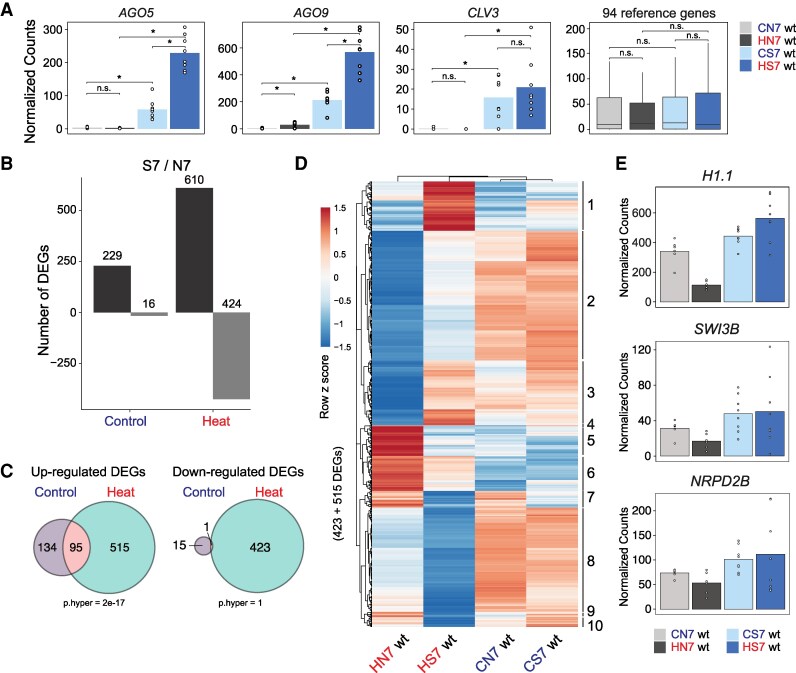
Heat response differs between stem cells and somatic cells. **A)** Normalized read counts for *AGO5*, *AGO9*, *CLV3*, and the average of 94 reference genes. C: control grown at regular temperature; H: heat-stressed; N: nonstem cell nuclei; S: stem cell nuclei; all samples from seedlings 7 d after germination. Statistical analysis was performed using a two-sided *t*-test, * indicates *P* < 0.05, n.s. indicates *P* > 0.05. Sample size: CN7 (*N* = 6), HN7 (*N* = 6), CS7 (*N* = 8), and HS7 (*N* = 8). In the boxplot, the boxes represent the interquartile range (IQR), the center line indicates the median, and the whiskers extend to 1.5 × IQR. **B)** Number of DEGs by pairwise comparison of stem cell at day 7 (S7) versus nonstem cells (N7). Black: upregulated genes; gray: downregulated genes. **C)** Venn diagrams with overlapping DEGs in **B)** for control versus heat-stressed samples. **D)** Clustered gene expression heat map. **E)** Normalized read counts for epigenetic regulators *H1.1*, *SWI3B*, and *NRPD2B*. Sample size as in **A)**.

We investigated the gene expression differences in wt stem cell versus nonstem cell nuclei directly after heat stress in more detail. The expression of 94 reference genes for stressed shoots (Czechowski et al. 2005) showed no differences between the samples (Fig. 3A). *AGO5* and *AGO9* are specifically expressed in stem cells (Bradamante et al. 2024), as also here confirmed by high enrichment in stem cells, together with stem cell marker *CLV3* (Fig. 3A). Heat stress even elevated *CLV3*, *AGO5*, and *AGO9* transcript levels in stem cell nuclei (Fig. 3A). In addition, the number of stem cell-specific differentially expressed genes (DEGs: Wald test FDR < 0.05 and log_2_ fold change > |1|) was more than doubled due to heat stress (Fig. 3B), with an increased expression of 515 genes and a decreased expression of 423 genes exclusively in stem cells (Fig. 3C). To identify expression patterns among these 938 (515 + 423) DEGs, we performed hierarchical clustering (Fig. 3D) and identified 10 major clusters (Fig. 3D, Supplementary Table S1).

To study if these 10 clusters represent functional groups, we performed a GO term analysis. Cluster 1 consists of genes with a strongly increased expression only in heat-stressed stem cells and is enriched for genes involved in “nucleic acid metabolic processes”, e.g. *MAG*, *SDN1*, and *DMR1*, important for DNA repair, small RNA metabolism, and DNA methylation (Santerre and Britt 1994; Ramachandran and Chen 2008; van Damme et al. 2009). Cluster 2 contains genes that are only slightly downregulated in stem cells after heat but much more down in nonstem cells, and these comprise gene categories “chromosome condensation” and “nucleosome assembly”. Among them are histone *H1.2* and subunit 2 of the condensin complex, suggesting less heterochromatin decondensation in stem cells during and after heat exposure. Interestingly, cluster 3 genes are strongly repressed in heat-stressed nonstem cells and include genes for maintaining shoot development and the cell cycle. This repression is not seen in stem cells and suggests that they maintain a cycling state and their developmental program even during intense heat, eventually facilitating recovery once the stress is over. Cluster 3 was also enriched for genes with negative regulatory roles. These genes comprised chromatin regulators, e.g. histone *H1.1*, *NRPD2B* (a PolIV subunit), and the chromatin remodeling subunit *SWI3B* (Fig. 3E, Supplementary Table S1). This indicates the increased resilience of stem cells to chromatin decompaction that otherwise occurs in nuclei of heat-stressed plants (Pecinka et al. 2010; Dumur et al. 2019). Genes of clusters 5 and 6 are characterized by strong upregulation in nonstem cells, but little change in stem cells and are enriched in photosynthesis-related genes. Genes of clusters 7 to 10 are characterized by strong downregulation in stem cells and are mostly enriched for innate immune and general stress response genes (Supplementary Table S1), further indicating that stem cells differ by suppressing stress-responsive pathways prevailing in other somatic cells.

### DNA methylation mutants differ in their heat response

**Figure 4.**
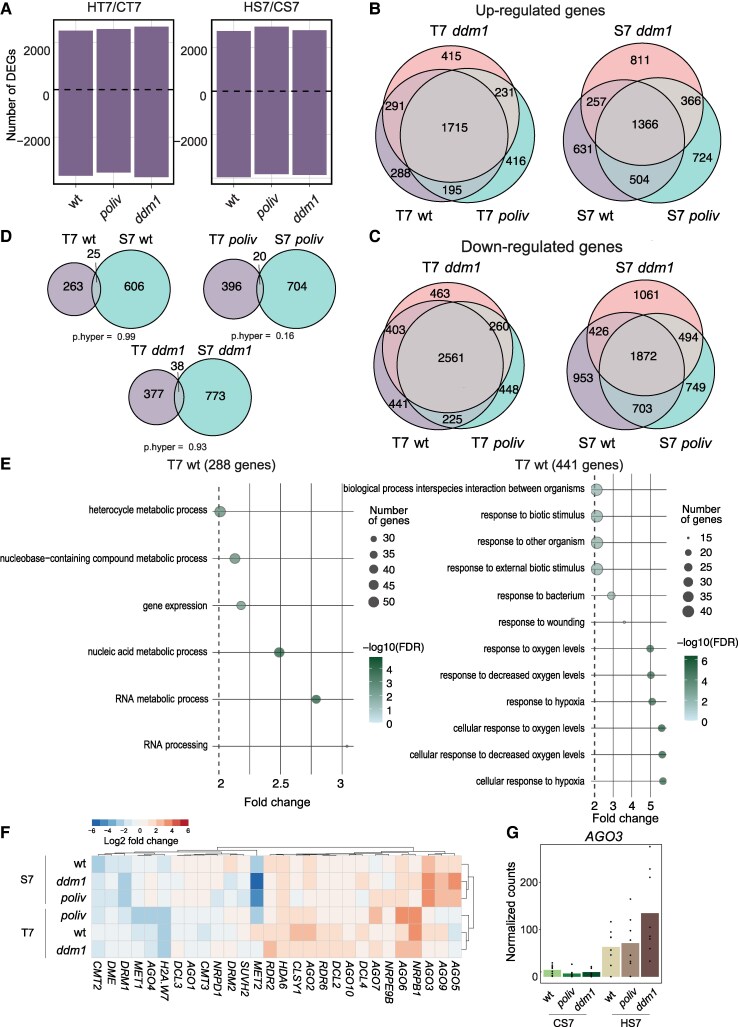
DNA methylation mutants differ in their heat response. **A)** Number of DEGs in seedlings (T for tissue) under heat stress compared with control conditions. wt = wild-type. Sample size: wt HT7 (*N* = 4), *poliv* HT7 (*N* = 4), *ddm1* HT7 (*N* = 4), wt CT7 (*N* = 4), *poliv* CT7 (*N* = 3), *ddm1* CT7 (*N* = 4), wt HS7 (*N* = 8), *poliv* HS7 (*N* = 8), *ddm1* HS7 (*N* = 8), wt CS7 (*N* = 8), *poliv* CS7 (*N* = 8), and *ddm1* CS7 (*N* = 8). **B,C)** Three-way Venn diagrams of upregulated **B)** and downregulated **C)** genes in wt, *poliv*, and *ddm1*. **D)** Venn diagrams for pairwise comparison of DEGs in stem cell nuclei (S7) and nuclei from whole seedlings (T7) in all 3 genotypes. Enrichment analysis was conducted using a hypergeometric test (p.hyper). **E)** Gene ontology analysis of distinctive upregulated and downregulated genes. **F)** Expression heatmap of TE regulators in stem cells (S7) and seedlings (T7). **G)** Normalized counts of AGO3 in stem cell nuclei under control (CS7) and heat stress (HS7). Sample size as in **A)**.

Next, we investigated the heat-induced gene expression differences between the wt and mutants with impaired DNA cytosine methylation only at CHH sites (*poliv*) or in all 3 sequence contexts (*ddm1*). Comparing the immediate heat response with controls in seedlings (HT) and stem cell (HS7) of wt, *poliv*, and *ddm1*, we identified more than 2,000 up- and over 3,000 downregulated genes for all 3 genotypes (Wald test FDR < 0.05 and log_2_ fold change > |1|, Fig. 4A).

Given the heat-hypersensitive phenotype shared between *poliv* and *ddm1*, we assumed that the expression of genes important for heat resilience and prevention of leaf necrosis should change either only in wt or in both *ddm1* and *poliv* seedlings. Therefore, we analyzed the overlap of gene expression changes (Fig. 4, B and C). The number of “private” DEGs for each genotype in seedlings ranged between 288 and 416 (up-DEGs) and 441 and 463 (down-DEGs). For the genes with heat-induced expression, these numbers were similar between stem cell nuclei and seedlings (Fig. 4B), but their overlap was not significant (Fig. 4D), underlining tissue- and genotype-dependent heat responses.

To understand which functional group of genes could be causative for the difference in heat resistance of seedlings, we performed GO term analysis of wt private DEGs (288 up-DEGs and 441 down-DEGs), and DEGs present only in *poliv* and *ddm1* seedlings (231 up-DEGs and 260 down-DEGs). For wt private DEGs, we found 6 significant GO terms for up- and 12 significant GO terms for down-DEGs (Fig. 4E). The GO terms for the upregulated genes described functions involved in RNA metabolism and included genes such as *CLSY1*, *AGO2*, and *DCL4*, which is crucial for the biogenesis of hairpin-derived and secondary 21 nt siRNAs, contributing to heat resistance (Fig. 4F) (Popova et al. 2013). This group also included the imprinted gene “*SUPPRESSOR OF DRM1 DRM2 CMT3*” *(SDC*), whose expression has been previously described to correspond with heat stress and recovery from heat stress (Sanchez and Paszkowski 2014). Interestingly, most genes of the downregulated DEGs belong to stress response pathways, except those related to heat stress response. This suggests that other defensive mechanisms were reduced to prioritize heat responses (Fig. 4E). GO terms of DEGs changing in *poliv* and *ddm1* also included mostly stress-related categories (Supplementary Fig. S5), and a loss of the homeotic balance between defense genes in these mutants could potentially contribute to increased heat sensitivity.

GO term analysis of private DEGs (Supplementary Fig. S6) revealed categories of DNA damage and repair for *ddm1* private DEGs (Supplementary Fig. S6, B and E) in seedlings and in stem cells, suggesting heat-induced genotoxic stress in *ddm1* by more frequent DNA breakage in decondensed chromatin or by increased transposon activity, which might also prevent recovery after the release of stress.

As we observed the upregulation of *AGO5* and *AGO9* as potential TE silencing factors, we also investigated which other host-counter defense genes against TE activation would become more active. Among genes known to be involved in TE silencing, we identified *AGO3* (Fig. 4G) as strongly induced by heat in stem cells in all 3 genotypes, even more pronounced in *poliv* and *ddm1* (Fig. 4F). AGO3 binds to transposon-derived small RNAs of 24 nt length (Zhang et al. 2016; Jullien et al. 2020) and, together with AGO5 and AGO9, could confer protection against TE activity in heat-stressed stem cells. Furthermore, we identified strong downregulation of *MET2* in stem cells (Fig. 4F), a homolog to the methyltransferase *MET1*, which is associated with TE copy number variation (Quadrana et al. 2016), though its mode of action is not yet clear. The expression of these genes did not change in seedlings, but *NRPB1* transcripts encoding the subunit of RNAPolII increased, whereas the expression of the heterochromatic histone variant *H2A.W7* (Lorkovic et al. 2017) decreased.

Taken together, we document notable differences in the heat response in stem cells versus seedlings, in all 3 genotypes tested. Furthermore, the overlap of private heat-induced DEGs between stem cells and seedlings is nonsignificant, suggesting that genes important for heat resistance in stem cells are different from those important in other tissues of seedlings.

### Some changes in gene expression persist beyond heat stress

**Figure 5.**
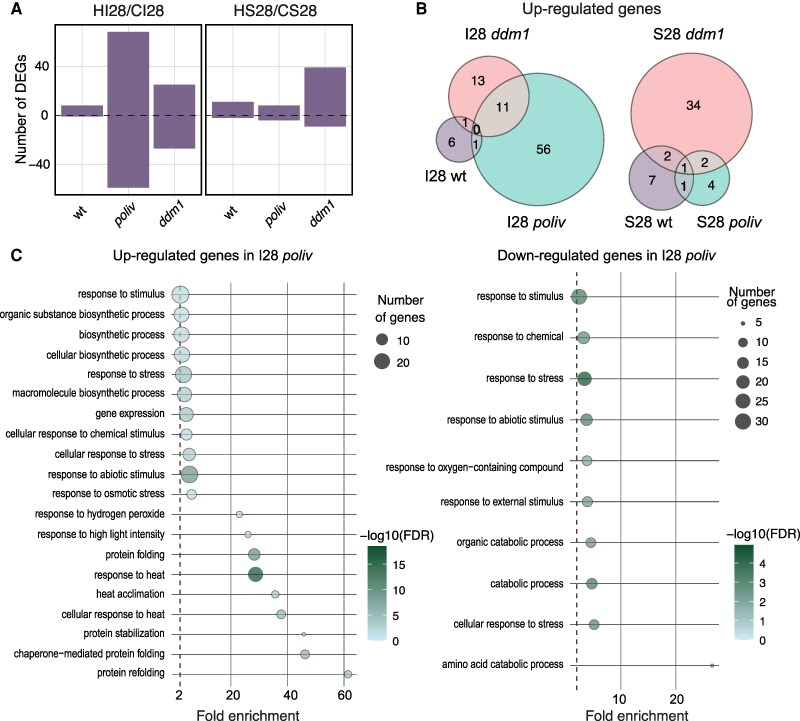
Persistent gene expression changes in *poliv* and *ddm1* after heat stress. wt = wild-type; C: control grown at regular temperature; H: heat-stressed; I: inflorescence meristem; S: stem cell nuclei; all samples from seedlings 28 d after germination. Sample size: wt CI28 (*N* = 3), *poliv* CI28 (*N* = 4), *ddm1* CI28 (*N* = 4), *wt* HI28 (*N* = 4), *poliv* HI28 (*N* = 4), *ddm1* HI28 (*N* = 3), wt CS28 (*N* = 4), *poliv* CS28 (*N* = 4), *ddm1* CS28 (*N* = 4), wt HS28 (*N* = 4), *poliv* HS28 (*N* = 4), and *ddm1* HS28 (*N* = 3). **A)** Number of DEGs in inflorescence meristem tissue (I28) and stem cell nuclei (CS28) 21 d after heat stress. **B)** Three-way Venn diagrams of upregulated genes in I28 and S28 in wt, *poliv*, and *ddm1*. **C)** Gene ontology analysis of upregulated and downregulated genes in I28.

Three wk after heat stress (28), the plants were mature and flowered, and all organs had developed post-heat stress. Correspondingly, gene expression differences were strongly reduced between control and heat-treated samples (Fig. 5A). Nevertheless, 11, 8, and 39 genes remained upregulated in stem cells (HS28) of wt, *poliv*, and *ddm1,* respectively (Fig. 5A, Supplementary Table S2). *APA1* (*AT1G11910*) that remained high in wt and *ddm1* had been shown to confer drought tolerance upon overexpression (Sebastián et al. 2020). Most genes upregulated in stem cells of *ddm1* were related to stress response (Supplementary Table S3), including the heat shock transcription factor *HSFC1* (*AT3G24520*) (Supplementary Table S2), indicating that *ddm1* stem cells still displayed stress response 3 wk after heat treatment.

Remarkably, we identified 25/68 up- and 33/55 downregulated heat-induced DEGs in inflorescence meristems (HI) of recovered plants in *poliv* and *ddm1*, respectively (Fig. 5B), in contrast to only 8 up- and 2 downregulated genes in wt. Similarly to HS28, many of these up-DEGs are related to stress response pathways, including response to heat (Fig. 5C). For *poliv*, 20 out of the 68 upregulated genes are involved in heat stress response, including the heat shock transcription factor *HFSA2* (*AT2G26150*). HSFA2 has previously been shown to drive a transcriptional heat stress memory (Charng et al. 2007; Friedrich et al. 2021) and directly activates many heat stress-responsive genes by recognizing heat-response elements (HREs) in their promoters (Schramm et al. 2006). This includes *ONSEN* and other TEs, and HSFA2 activity could be the main reason for the increase of TE expression upon heat stress (Pietzenuk et al. 2016). This supports the importance of DNA methylation for a quick recovery after heat stress and might contribute to the increased heat sensitivity of *poliv* and *ddm1* mutants.

### Transposons can remain active after the heat stress

**Figure 6.**
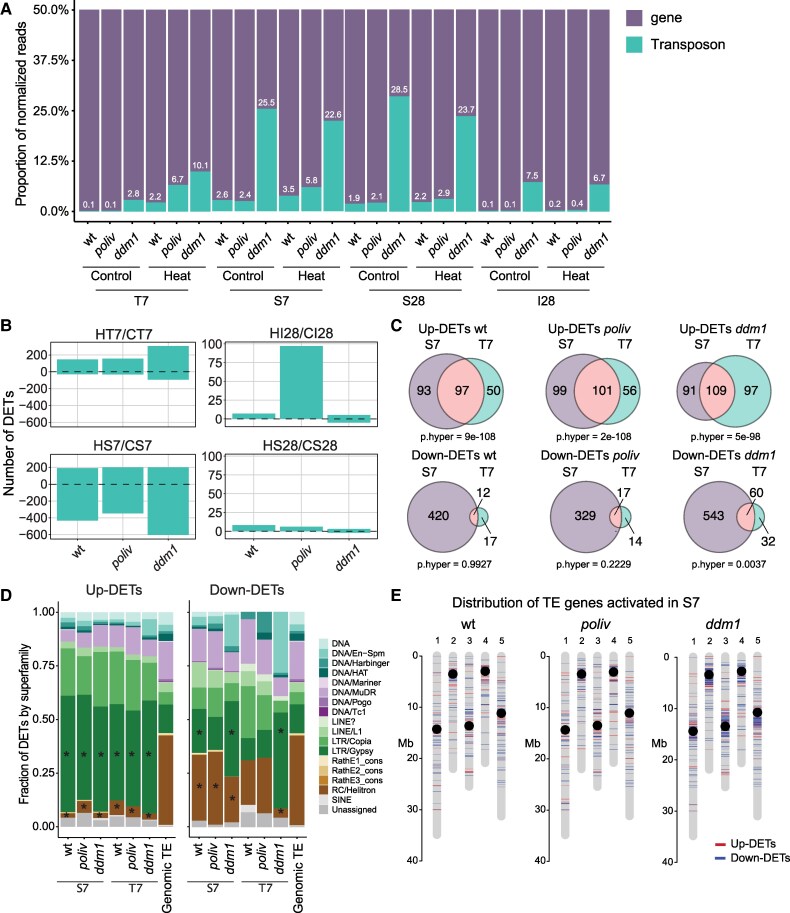
Expression of TEs in response to heat stress. **A)** Fraction of genes and TEs by normalized counts. Sample size as in Figs. 4 and 5. **B)** Number of up- or downregulated TEs by tissues: T7: seedling; I28: inflorescence meristem; S7: stem cell at D7; S28: stem cell at D28. Sample size as in Figs. 4 and 5. **C)** Venn diagrams indicate overlaps of up- and down-DETs between HS7/CS7 and HT7/CT7 for the 3 genotypes. **D)** Fraction of DETs classified by superfamilies. Genomic TE: Proportion of the families in the reference genome. Enrichment analysis was performed using a hypergeometric test (p.hyper), * indicates *P* < 0.05. **E)** Distribution of TE genes activated in HS7 along the 5 chromosomes.

Extending our analysis to TE expression, we quantified the proportion of reads aligning to genes versus TE sequences (Fig. 6A). As expected, this ratio is higher in *ddm1* than in the other 2 genotypes independent of the conditions, due to the much higher baseline of TE activity in this mutant (Hirochika et al. 2000). Nonetheless, in seedling tissue (HT), TE reads increased in all 3 genotypes upon heat (Fig. 6A). We also observed increased TE transcript ratios in stem cell nuclei of wt and *poliv* after heat treatment (HS7), later (HS28), and in inflorescence meristems (HI), but in *ddm1* samples, the proportion was slightly lower at both time points and in meristems.

To identify individual TEs that change expression upon heat, we calculated differentially expressed TEs (DETs, Wald test FDR < 0.05 and log_2_ fold change > |1|) of heat-stressed versus control samples for all genotypes (Fig. 6B). The number of upregulated DETs was similar between stem cell nuclei and seedlings, approximately 190 TEs (Fig. 6B), with a significant overlap of heat-induced DETs in stem cells compared to seedlings (Fig. 6C). In addition, 91 and 76 TEs were upregulated independently of the genotype in seedlings and stem cells, respectively (Supplementary Fig. S7A). Of those, 54 showed increased expression in stem cells and seedlings (Supplementary Fig. S7C). These 54 TEs contained all 8 *ONSEN* copies and consisted mainly of LTR/Copia and LTR/Gypsy TEs (Supplementary Table S4); 23 of them were larger than 4 kb and candidates for potentially autonomous elements. Remarkably, the overlap between downregulated TEs is minimal, and the numbers are different: only 7 in seedling tissues compared to 129 in stem cells (Supplementary Fig. S7, B and D). In contrast to the family specificity among upregulated TEs in stem cells and seedlings, the superfamily distribution of downregulated TEs was more similar to the genomic distribution and contained more DNA TEs (Fig. 6D). Only TEs downregulated in *ddm1* were also enriched for LTR/Gypsy elements (Fig. 6D).

Loss of DDM1 results in loss of DNA methylation, primarily at long heterochromatic TEs near centromeres (Zemach et al. 2013), regions that are decondensed by acute heat stress (Pecinka et al. 2010; Dumur et al. 2019). Therefore, we asked for the chromosomal localization of heat-induced up- and downregulated DETs and found them distributed all over all chromosomes in heat-stressed wt and *poliv*, whereas DETs downregulated in *ddm1* are enriched at the pericentromeres (Fig. 6D), concordantly with the enrichment for LTR/Gypsy TEs (Underwood et al. 2017) (Fig. 6E).

**Figure 7.**
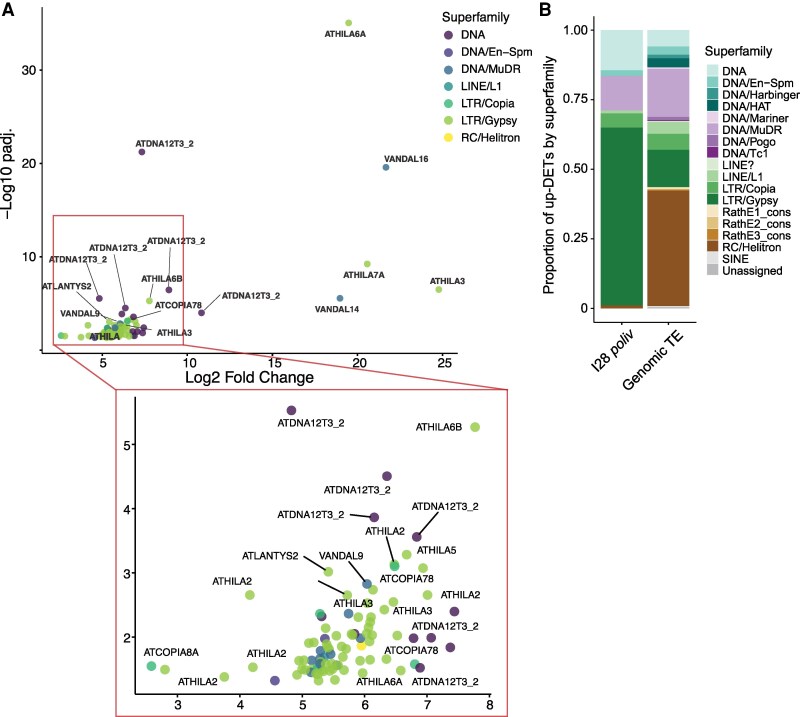
Persistent TE expression changes after heat stress. **A)** Up-DETs of 97 TEs in inflorescence (I28) of *poliv*. **B)** Fraction of TE superfamilies of 97 up-DETs in inflorescences of *poliv*.

Intriguingly, we found 97 TEs still upregulated in the inflorescence tissue of D28 *poliv* (Fig. 6B), including 2 members of *COPIA78* (*ONSEN*, *AT3TE92525*, and *AT5TE15240*) (Fig. 7A). We also detected highly increased expression (log_2_ FC > 7.5) of 4 LTR/Gypsies, 2 DNA/MuDRs, and 2 DNA TEs (Fig. 7A). Most of these TEs belong to LTR/Gypsy and LTR/Copia elements (Fig. 7B), and 65 out of the 97 TEs contained a potential HSFA2 binding motif (Supplementary Table S5). The discrepancy in TE expression between I and S28 in *poliv* is likely due to the presence of cytoplasmatic RNA in meristem samples, while this is absent in stem cell nuclei pools. Extrachromosomal copies of retrotransposons (LTR/Gypsy and LTR/Copia) could also be templates for polymerases and could persist for long periods after activating heat stress. The persistent upregulation of *HSFA2* in *poliv* meristems after 3 wk of heat exposure likely contributes to the activation of heat-responsive TEs.

### DNA methylation changes in stem cells can persist beyond heat stress

**Figure 8.**
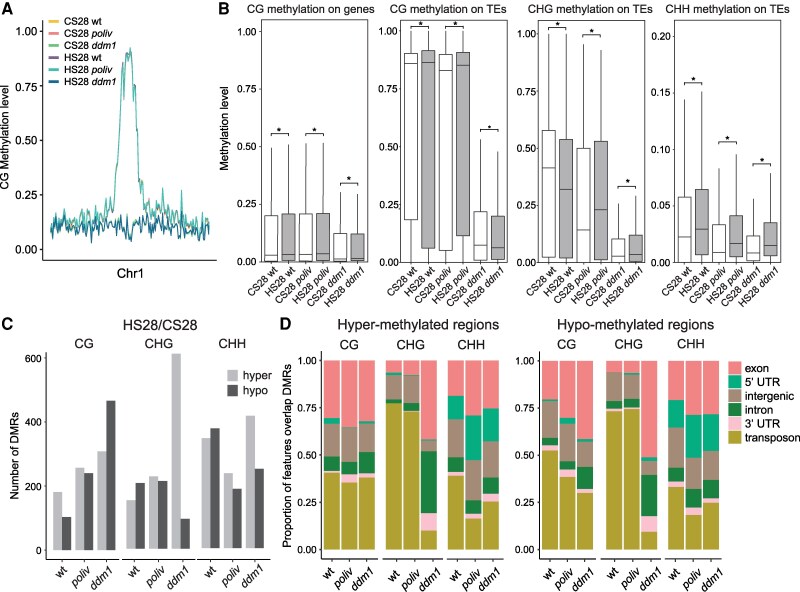
Heat stress triggers long-lasting DNA methylation changes in SAM stem cells of inflorescences. **A)** Relative CG methylation across chromosome 1, quantified in 200 kb windows. **B)** Relative methylation levels for CG sites at genes, CG/CHG/CHH sites at TEs. HS28 indicates stem cells from plants heat-stressed 3 wk earlier; CS28 indicates stem cells from mock-treated control plants. Statistical analysis was performed by Wilcoxon test, * indicates *P* < 0.05. **C)** DMRs at D28. **D)** Hyper- and hypomethylation in different sequence contexts sorted according to different genomic features.

As described, DNA methylation is a central element in the control of TE activity. Changes in this modification at TE sequences have been associated with various stresses (e.g. Wibowo et al. 2016; Korotko et al. 2021). As part of the epigenetic information, DNA methylation changes can persist during several cell division cycles and influence phenotypes (Quadrana and Colot 2016; Wibowo et al. 2016). Loss of components maintaining DNA methylation leads to heritable hypomethylation (Saze et al. 2003; Johannes et al. 2009; Reinders et al. 2009). For stress-induced changes in methylation patterns to become heritable to the next generation, DNA methylation changes must pass through the stem cells that form the germ line. To investigate whether heat stress-induced methylation changes would persist in stem cells, we sampled them for bisulfite sequencing from heat-stressed and control wt, *poliv*, and *ddm1* plant 3 wk after treatment. Large-scale DNA methylation patterns were however similar between heat-treated and control samples (Fig. 8A). As expected, TE DNA methylation in *ddm1* stem cells was low in all sequence contexts and reduced at CHG and CHH in *poliv* (Fig. 8B). Slight differences at CHG sites of TEs in heat-stressed stem cells, decreased in wt but increased in *poliv* (Fig. 8B) were not significant and likely due to interference of heat-induced chromatin decondensation with the activities of CMT2, CMT3, and RdDM.

However, employing a two-state hidden Markov model (HMM) (Hüther et al. 2022), we identified differentially methylated regions (DMRs), comprising hundreds of heat-induced hypo- and hyper-DMRs in CG, CHG, and CHH contexts in all 3 genotypes (Fig. 8C). wt showed more hyper- than hypo-DMRs at CGs but less in CHG and CHH contexts. *poliv* had similar numbers of hyper- and hypo-DMRs in CG and CHG contexts but slightly fewer hypo-DMRs in the CHH context. Interestingly, *ddm1*, with generally low methylation levels in all sequence contexts, displayed the highest numbers of heat-induced DMRs, with less hyper- than hypo-DMRs in CG, but considerably more hyper- than hypo-DMRs in CHG and CHH contexts. Most DMRs overlapped with transposons or genic regions, a few with intergenic sequences (Fig. 8D). CG-DMRs overlapped mostly with transposon or exonic sequences. CHH-DMRs overlapped additionally to a large extent with 5′UTRs. Most DMRs in the CHG context in wt and *poliv* overlapped with transposons, in contrast to *ddm1* CHG-DMRs that were mainly in exonic and intronic sequences.

Asking for a significant overlap of DMRs with genes that were still deregulated at D28 (Supplementary Table S6), we found only 4 hypo-DMRs overlapping with 2 genes and 4 TEs with increased expression in HI28 *poliv*, but almost no hyper-DMRs at less expressed genes and TEs at D28. However, even without a direct link to changed expression levels, the presence of hundreds of DMRs in SAM stem cells even after 3 wk of recovery from severe heat stress indicates a window of opportunity for lasting effects. Their occurrence seems to depend to some extent on the different preexisting DNA methylation states in wt and the mutants. However, these DMR sites in the stem cells do not significantly overlap with DMRs identified after different stress treatments in other tissues (Wibowo et al. 2016; Ichino et al. 2021) (Supplementary Table S7). Whether the DMRs occur arbitrarily or at specific sites, whether they are reset during germline differentiation, or can potentially be inherited, remains to be investigated.

## Discussion

Numerous studies have investigated the reaction of plants to adverse elevated temperatures, revealing a complex network of signaling and regulatory components dampening the deleterious effects (recently reviewed by Zhao et al. (2020), Zioutopoulou et al. (2021), Zhou et al. (2022), and Kan et al. (2023)). Beyond the immediate response, acclimation and priming can contribute “memory” effects that help upon continuing or repetitive heat stress (Perrella et al. 2022; Hemenway and Gehring 2023; Kappel et al. 2023; Nishio et al. 2024). How long-lasting such adaptive changes are, and whether they can be transmitted to progeny, is often not clear. However, undisputed evidence for inherited effects of heat stress is new insertions of TEs found after heat exposure of the parental plants (Ito et al. 2011; Matsunaga et al. 2015; Papolu et al. 2022). Their potential to create genetic variation has likely contributed to evolutionary stress adaptation, but only if occurring in those cells that contribute to the germline. The same applies to spontaneous or induced epigenetic changes. Studies of heat stress effects were mostly made by analyzing whole seedlings, organs, or excised tissue. The experiments presented here analyze heat stress effects specifically in the stem cells of the SAM, from which all generative cells originate. The reporter construct labeling the nuclei of the stem cells in wild-type background and introgressed in mutants defective in 2 major epigenetic transposon control pathways allowed transcriptome and methylome analyses, comparing stem with nonstem cells, from control plants, plants with profound but sublethal heat stress, and plants recovering from these conditions.

Not surprisingly, heat stress causes substantial changes in the transcriptome of all samples and a global return to pre-stress states after recovery. During nuclei extraction, we realized that a lower number of nuclei is extractable after heat shock; however, this survival bias should be independent of the tissue type from which the nuclei originate.

Stem cells differ from nonstem cells in their response in specific ways, showing unique patterns in both up- and downregulated DEGs. Stress-related genes, more lowly expressed in stem than nonstem cells, and developmental genes more highly expressed in the stem cell samples point to different “priorities”: the stem cells do not activate the stress response but keep their potential for growth and development, which might prepare these cells for a quick recovery once the stress is over. This hypothesis is in line with the concept of an autonomous heat stress memory, based on a study with gene expression in manually excised meristems (Olas et al. 2021). As in our previous study (Gutzat et al. 2020; Bradamante et al. 2024), stem cells were enriched for *AGO5* and *AGO9* transcripts. Their expression was even further increased in heat-stressed samples, together with *AGO3*, another member of the family encoding transposon-derived sRNA-binding proteins. Although the respective Arabidopsis mutants do not show obvious TE de-repression under nonstress conditions (Jullien et al. 2020; Bradamante et al. 2024), *AGO5* orthologs in maize control the activity of retrotransposons during male gametogenesis, and mutants have strongly reduced male fertility under heat stress (Lee et al. 2021). This suggests a potential and likely complementary role of AGOs in TE repression that may become only evident, or relevant, during stress conditions that increase TE activity. The role of epigenetic factors for heat tolerance is also well documented beyond transposon control (Popova et al. 2013; Zioutopoulou et al. 2021; Ramakrishnan et al. 2022; Nishio et al. 2023). Especially, DNA methylation has been shown to correlate with temperature and geographic origin of different Arabidopsis accessions (Dubin et al. 2015). It was therefore not unexpected that the 2 mutants with decreased DNA methylation included in our study turned out to be more heat-sensitive than the relatively resilient wild-type Col-0, although *ddm1* and *poliv* were not identified as heat-sensitive in a previous study with soil-grown plants (Popova et al. 2013), likely due to differences in growth conditions and plant developmental stages at the time of stress exposure. Also expected were the distinct clusters between wt and *poliv* transcriptional profiles on one side and that of *ddm1* at the other, as the much more drastic loss of DNA methylation in *ddm1* is well documented to change transcription of transposons and protein-coding genes even independent of stress in Arabidopsis and rice (Tan et al. 2016; Lee et al. 2023). Heat-induced changes in our data did not override these genotype-specific differences but overlapped to a large extent. However, although DNA methylation seems to be an important and central component, it must be seen in connection with other chromatin-related factors. These are, among others, its feedback regulation with core histone modification (reviewed in Law and Jacobsen (2010)) or its interplay with the linker histone H1 (Zemach et al. 2013; Choi et al. 2021; Liu et al. 2021). Stem cell-specific analysis of these additional factors is needed in the future to disentangle how far these cells differ in their epigenetic setup from surrounding somatic cells.

Stress response is costly, and the reversion of most transcripts to the pre-heat levels 3 wk after return to regular temperature confirms a quick recovery. The exceptions of persistent changes could have been due to RNA features that would delay degradation. However, a higher number of still upregulated genes in the 2 mutants compared to the wt, and an enrichment of stress-related genes suggest rather a functional selection. Noticeable is the elevated level in the *poliv* mutant of the transcript encoding HSFA2, a heat stress response factor involved in the activation of *ONSEN* transposon with an HRE element in its promoter (Cavrak et al. 2014). *poliv* is the mutant in which new insertions were found in the next generation of heat-stressed parental plants (Ito et al. 2011). Therefore, higher levels of HSFA2 in stem cells and an accumulation of *ONSEN* in the meristem (Cavrak et al. 2014) could have contributed to higher chances of new insertions during formation of the germ line and is in line with more insertions upon defects in the initiating part of the RdDM pathway (Hayashi et al. 2020). However, it must be kept in mind that there are RdDM-independent mechanisms leading to new insertions (Masuta et al. 2017). In addition, the interesting finding that one burst of transposition precludes a second one in the same cell lineage, together with a preference for genic insertions that are likely not interfere with the development of germline and gametes (Gaubert et al. 2017) indicates a sophisticated interplay between genetic and epigenetic factors of host and genetic parasite. As for the exceptional persistence of transcripts during the recovery period, the findings of hundreds of regions at which heat-induced changes in DNA methylation remained within our experimental timeframe are likely part of this relationship. The lasting DMRs in the stem cells were not directly connected with the DEGs or transposons but might represent “windows of opportunities”. Whether they are due to the activity of writers or erasers under stress conditions, passive or arbitrary consequences of drastic genome rearrangements during heat stress, or persist into the next generation, remains to be investigated. Again, DNA methylation is just one component of potentially heritable information that is influenced by heat stress, as indicated by the role of HSFA2 as part of a heat stress memory, where it is required to deposit activating histone modifications (Lämke et al. 2016). Drastic changes in chromatin accessibility and composition reshape the transcriptional landscape between generations regularly, but are likely influenceable by external conditions (Borg et al. 2021; Hemenway and Gehring 2023; Nishio et al. 2023). Therefore, our work is only a starting point to differentiate between transient, long-lasting, and permanent consequences of stress. However, the methodology to analyze the specific population of stem cells, the observed differences between them and other somatic cells, and their role as germ line precursors invite further studies on whether epimutations occur randomly, whether and when they are reset, and whether they lead to permanent gene expression changes. If so, and if they consequentially result in phenotypic effects, they might be relevant, for natural evolution or for breeding purposes.

## Materials and methods

### Plant material and growth conditions

*Arabidopsis thaliana* ecotype *Col-0* was used for all experiments. The stem cell reporter *pCLV3::H2BmCherry* is described in Gutzat et al. (2020) and here referred to as “wt”, in contrast to the mutants that carry the same reporter after combining it by crossing with *poliv* (*nrpda-3—*SALK_128428) or *ddm1* (*ddm1-10*—SALK_093009). Plants were grown either in vitro on GM medium or soil, with 16/8 h light/dark cycles. Control plants were consistently grown at 21 °C; for heat exposure, we applied 37 and 44 °C for 24 or 48 h.

### Description of samples

An overview of the experimental procedures and sample preparation is presented in Supplementary Fig. S1. Six-day-old seedlings grown in vitro were subjected to either heat exposure (H) or mock treatment as control (C) for 24 h. Following treatment, the seedlings were processed for RNA extraction and transcriptome analysis. For the wild-type, 2 types of samples were prepared: (i) seedling tissue (T), which included whole seedlings without roots, and (ii) nuclei from stem cells (S) and nonstem cells (N), isolated from hand-dissected meristem tissue and separated by fluorescence-activated nuclei sorting (FANS).

Control and heat-stressed seedlings grown in parallel, including the mutants with the stem cell marker, were transferred to soil and allowed to grow for an additional 21 d. At 7 + 21 = 28 d, the tips of the inflorescences containing the shoot apex and inflorescence meristems were dissected. This inflorescence tissue (I) was either processed for RNA extraction or subjected to FANS to isolate stem cell nuclei. These nuclei were then used for RNA and DNA extraction to analyze and compare mRNA levels and DNA methylation patterns.

### DNA extraction

To prepare DNA for methylation analysis by bisulfite sequencing, 5000 FANS-ed nuclei were used to extract DNA with the Quick-DNA microprep kit (Zymo Research #D3020).

For Southern blots, DNA was extracted from 300 mg of above-ground tissue of seedlings with a cetyltrimethylammonium bromide (CTAB)-based method adapted from https://opsdiagnostics.com/notes/protocols/ctab_protocol_for_plants.htm. In short, frozen tissues were ground and transferred to 1.5 mL CTAB buffer with 1% PVP and 1% RNase and incubated at 60 °C for 30 min. The tubes were centrifuged twice for 10 min at 14,000 *g* to remove debris. Supernatants were transferred to new tubes containing chloroform/isoamyl alcohol, carefully mixed, and centrifuged at 10,000 *g* for 2 min. The aqueous upper phases were transferred, mixed with 0.7 volume of isopropanol, and incubated at −20 °C overnight. The samples were centrifuged at 14,000 *g* for 15 min, the supernatants were removed, and the pellets washed twice with 70% ethanol. The pellets were dried under a sterile hood for 5 min and resuspended in 70 *µ*L TE.

### RNA extraction

Ten dissected seedlings (T) or 10 inflorescence meristems (I) were used for trizol RNA extraction adapted from Rio et al. (2010). Frozen tissues were ground and mixed with 375 *µ*L of TRI agent and incubated on ice for 10 min. Next, 100 *µ*L of chloroform was added and vigorously vortexed. The tubes were centrifuged for 10 min at 14,000 *g* and the aqueous phases were transferred to new tubes containing 1 volume of isopropanol and 1 *µ*L of RNA-grade glycogen (ThermoFisher #R0551). The mix was incubated at −20 °C overnight. The tubes were centrifuged, and pellets were washed twice with 70% ethanol and dried for 5 min. The pellets were dissolved in 50 *µ*L nuclease-free water.

### Southern blots for the detecting of ONSEN extrachromosomal DNA

For Southern blot detection, 10 *µ*g of genomic DNA was restricted by *Hpa*II (ThermoFisher # ER0511) overnight. Afterward, the enzyme was inactivated by incubating the tubes at 95 °C for 5 min. DNA was precipitated by adding 3 volumes of ethanol, 1/10 volume of 3 m NaAc pH 5.2, and 1 *µ*L of glycogen and incubated at −20 °C overnight. The mixtures were centrifuged at 14,000 *g* for 15 min, the supernatants removed, and the pellets were washed twice with 70% ethanol. The DNA pellets were eluted in 20 *µ*L TE buffer. After adding the gel loading solution, the samples were run on 1% TAE agarose gels.

After sufficient separation, the gels were rinsed with 250 mm HCl for 10 min, then with denaturation solution (500 mm NaOH, 1.5 m NaCl) for 30 min, and incubated in a neutralization buffer (0.5 m Tris, 1.5 m NaCl, 1 mm EDTA) for 30 min. DNA was transferred onto a Hybond NX membrane (Merck #GERPN203T) and cross-linked by a UV linker (Stratagene UV Stratalinker 2400).

The probe was generated by PCR amplification of the *ONSEN* sequence with forward primer TCTAGAATCATCTTCCACCTCCTTA and reverse primer ATCCTTGATAGATTAGACAGAGAGCT. The resulting amplicon was labeled with α-^32^P-dCTP using the labeling kit (Aligent #300385). The membranes were hybridized with the denatured probe at 65 °C overnight in hybridization buffer (0.4 m Na_2_HPO_4_, 0.1 m NaH_2_PO_4_, 20% SDS, 0.5 mm EDTA) and exposed to a phosphor screen for 1 d and imaged by phosphor-imager (Amersham Typhoon).

### Fluorescence-activated nuclei sorting (FANS)

FANS was adapted from a protocol described before (Gutzat and Mittelsten Scheid 2020; Bradamante et al. 2024). Briefly, 200 mg of above-ground seedlings at D7 and inflorescence meristems at D28 were collected in ice-cold Galbraith buffer (Galbraith et al. 1983) (45 mm MgCl_2_, 30 mm sodium citrate, 20 mm MOPS, 0.1% triton, 0.5% 2-mercaptoethanol), Ribolock RNase Inhibitor (1 U/*µ*L Thermo Fisher Scientific #EO0382), and protease inhibitor cocktail added (Roche #06538282001) according to manufacturer's indication. To isolate nuclei, tissues were disrupted using a TissueRuptor (Qiagen #990890) for 1 min at 5,000 rpm, and debris was filtered (Sysmex #04-0042-2316). After filtering, the suspension was centrifuged at 1,500 *g* for 10 min, and pellets were washed twice with 1 and 2 mL Galbraith buffer, respectively, containing 5 *µ*g/mL DAPI (Sigma #D9542-1MG). Nuclei were sorted according to the detection of mCherry and DAPI fluorescence on a BD FACSAriaTM III Cell Sorter (70 *μ*m nozzle). Gates were adjusted using nontransgenic Col-0 nuclei as reference.

### mRNA and bisulfite library preparation and sequencing

From FANS-sorted material, bulks of 100 nuclei for each replicate were used for library preparation. Smart-seq v2 (seedling, inflorescence meristem, stem cell D28) and v3 (stem cell D7) library preparation and sequencing were performed by the Next Generation Sequencing Facility (Vienna BioCenter Core Facility) NovaSeq SP SR100. For bisulfite sequencing, libraries were prepared with the Pico Methyl-Seq prep kit (Zymo Research #D5456) and sequenced by the Next Generation Sequencing Facility (Vienna BioCenter Core Facility) NovaSeq SP SR100.

### RNA-Seq analysis

The analysis strategy is explained in detail in Bradamante et al. (2024). With the release of the Arabidopsis genome annotation TAIR8, a transposon annotation based on multiple homology-based predictions has been integrated (Buisine et al. 2008). Existing annotations that overlap with TE annotations have been reclassified under the locus type “transposable element gene” (https://arabidopsis.org/download_files/Genes/TAIR8_genome_release/Readme-transposons). This introduces an issue of redundant annotations when aligning and assigning sequencing reads to genes, transposons (TEs), or TE genes. To prevent the resulting overlap between TE genes and TEs, we excluded TE genes from the TAIR10 annotation and incorporated TEs as a single feature type (TAIR10 + TEs). Raw bam files were converted to fastq by bedtools (v2.27) (Quinlan and Hall 2010). The fastq files were used as input for the nextflow rnaseq pipeline (v21.02.0) (Ewels et al. 2020) with additional parameter “--clip_r1 19 --clip_r2 9 --three_prime_clip_r1 5 --three_prime_clip_r2 5”. Output count files were used to analyze differential gene and TE expression by DESeq2 (Love et al. 2014) with filtering out loci with less than 5 reads per genotype on average and with the cutoff of *p.adj*. < 0.05, log_2_ fold change > |1|. Gene ontology enrichment was performed using the PANTHER classification system (https://geneontology.org). Visualization of the data was done with R using the packages tidyverse, ggplot2 (Wickham 2016), pheatmap (Kolde 2018), and chromplot (Oróstica and Verdugo 2016).

### Bisulfite-seq analysis

Raw bam files were converted to fastq by bedtools (v2.27) (Quinlan and Hall 2010). Adaptors were removed with trim galore (v0.6.2) (Krueger and Andrews 2011) with parameter --clip_r1 13 --three_prime_clip_r1 12. Genome alignment was performed with bismark (v0.22.2) (Krueger and Andrews 2011) with bowtie2. Output files were deduplicated by deduplicated_bismark (bismark v0.22.2). The methylation level was obtained from methylpy call-methylation-state (v1.2.9) (Schultz et al. 2015). DMRs were obtained using a two-state HMM method on nextflow methylscore (v21.10.06) (Hüther et al. 2022).

## Supplementary Material

kiaf110_Supplementary_Data

## Data Availability

Sequence data from this article can be found in the Gene Expression Omnibus database at https://www.ncbi.nlm.nih.gov/geo/, accession number GSE223915. Accession numbers of genes mentioned in the text are listed in Supplementary Table S8.

## Supplementary data

The following materials are available in the online version of this article.

**Supplementary Figure S1.** Schematic description of sample preparation.

**Supplementary Figure S2.** Analysis of *ONSEN* extrachromosomal DNA quantified through Southern blot hybridization (extended from Fig. 1B).

**Supplementary Figure S3.** Collection of stem cell nuclei marked with fluorescence proteins.

**Supplementary Figure S4.** Principal component analysis (PCA) of mRNA data, separated by chronological age of the samples.

**Supplementary Figure S5.** Gene ontology analysis of uniquely up- and downregulated genes in seedlings.

**Supplementary Figure S6.** Gene ontology analysis of various gene sets.

**Supplementary Figure S7.** Venn diagrams illustrating differentially expressed transposons (TEs) in seedlings (T7) and stem cells at day 7 (S7).

**Supplementary Table S1.** Differentially expressed genes and GO term analysis.

**Supplementary Table S2.** Differentially expressed genes at D28.

**Supplementary Table S3.** Gene ontology enrichment of upregulated genes in SC7 ddm1.

**Supplementary Table S4.** Heat-induced TEs independent of geno- and tissue type.

**Supplementary Table S5.** Potential TEs containing HSF binding sites.

**Supplementary Table S6.** Overlaps between differentially expressed genes/TEs and differentially methylated regions.

**Supplementary Table S7.** DMRs overlapping with hypervariable DMRs.

**Supplementary Table S8.** List of gene names and accession numbers.

## Dive Curated Terms

The following phenotypic, genotypic, and functional terms are of significance to the work described in this paper:

CMT2 Gramene: AT4G19020
CMT2 Araport: AT4G19020

CMT3 Gramene: AT1G69770
CMT3 Araport: AT1G69770

DCL3 Gramene: AT3G43920
DCL3 Araport: AT3G43920

RDR2 Gramene: AT4G11130
RDR2 Araport: AT4G11130

TRI Gramene: AT2G29330
TRI Araport: AT2G29330

AGO5 Gramene: AT2G27880
AGO5 Araport: AT2G27880

CLV3 Gramene: AT2G27250
CLV3 Araport: AT2G27250

NRPD2B Gramene: AT3G18090
NRPD2B Araport: AT3G18090

AGO2 Gramene: AT1G31280
AGO2 Araport: AT1G31280

MET1 Gramene: AT5G49160
MET1 Araport: AT5G49160

DDM1 Gramene: AT5G66750
DDM1 Araport: AT5G66750
