# Supplementary material for: Heat stress response and transposon control in plant shoot stem cells
Source: Plant Physiol. 2025 Mar 28;197(4):kiaf110. doi: 10.1093/plphys/kiaf110 (PMC11997658; doi:10.1093/plphys/kiaf110)
Supplement: kiaf110_Supplementary_Data [file kiaf110_supplementary_data.zip › Supplementary Data.pdf]

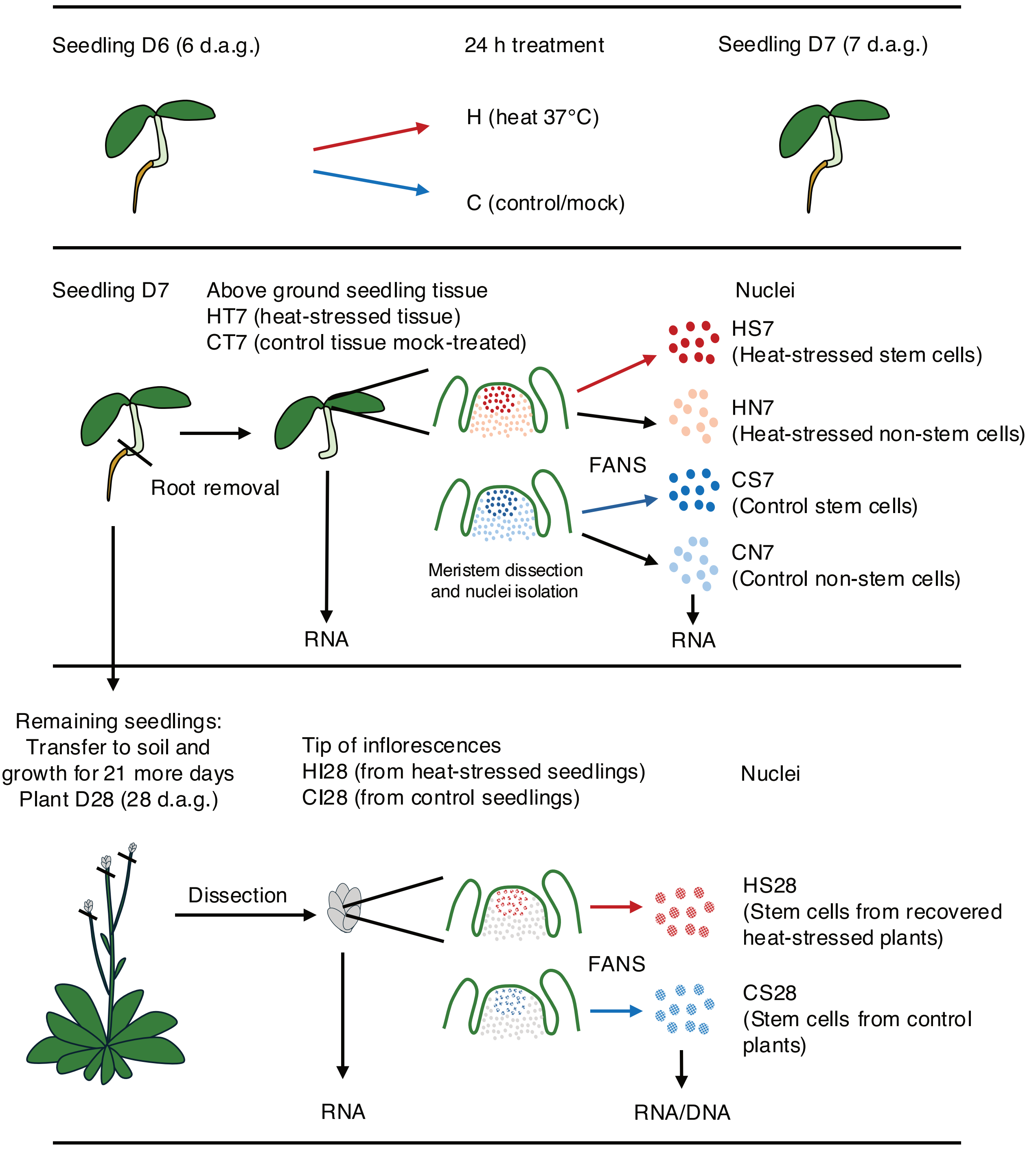

Supplemental Figure S1. Schematic description of sample preparation

d.a.g.: days after germination; FANS: fluorescence-activated nuclei sorting; RNA: transcriptome analysis; DNA: DNA methylation analysis. Genotypes in the study: Col-0 (accession background of all strains); wt (*pCLV3::H2BmCherry* in Col-0); *poliv* (*pCLV3::H2BmCherry* in *nrpda-3* - SALK_128428); *ddm1* (*pCLV3::H2BmCherry* in *ddm1-10* - SALK_093009).

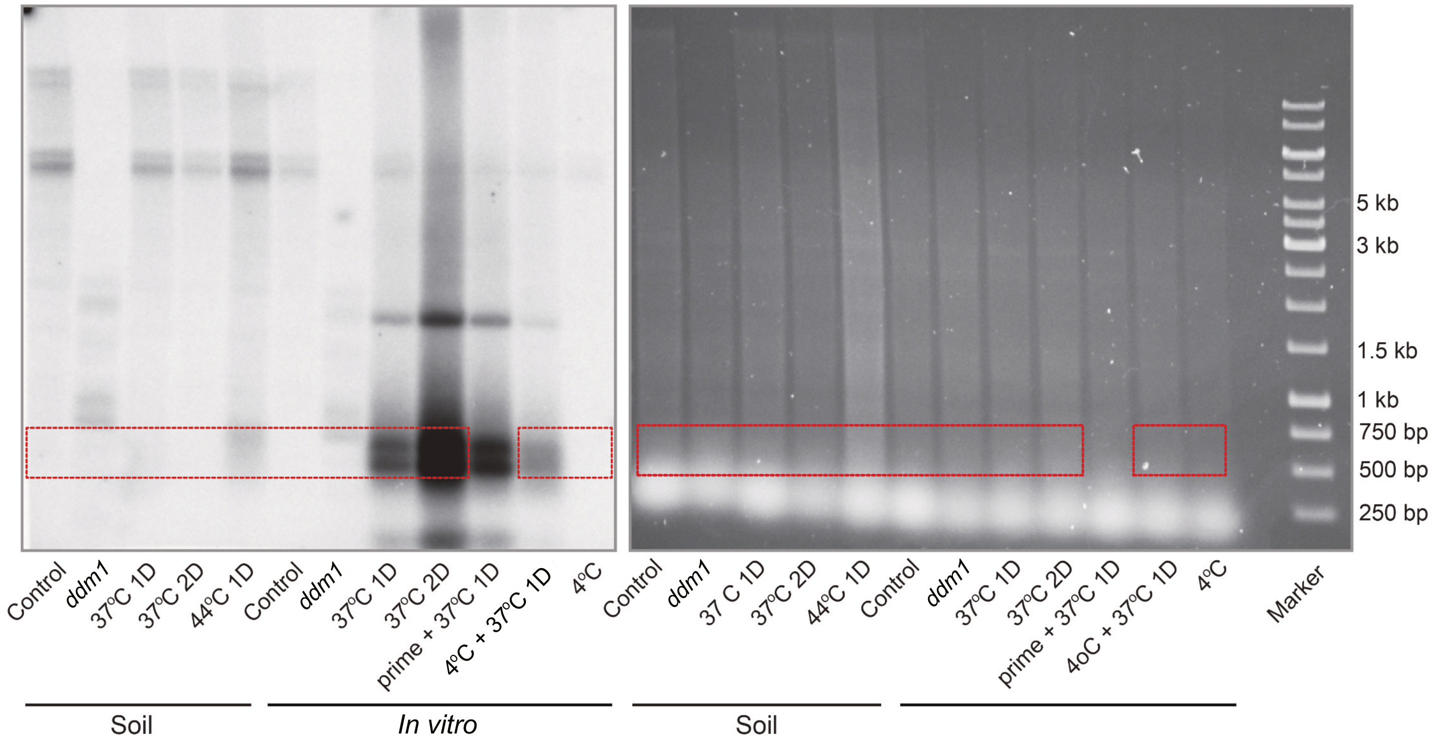

Supplemental Figure S2. Analysis of *ONSEN* extrachromosomal DNA quantified through Southern blot hybridization (extended from Figure 1B). Each lane indicates the culture medium, temperature, and heat stress duration. All samples correspond to wild type plants, except for *ddm1*. The red boxes indicate blot and gel sections shown in Figure 1B.

A

Control condition

Col-0

Tube: col-0

Population	#Events	%Parent	%Total
All Events	400,000	####	100.0
P1	386,087	96.5	96.5
P2	370,409	95.9	92.6
P3	110,468	29.8	27.6
P4	63,690	57.7	15.9
neg	30,902	48.5	7.7
P9	20,573	66.6	5.1
P5	4	0.0	0.0

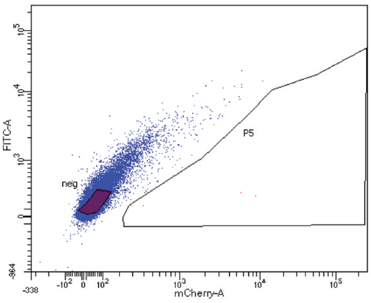

pCLV3::H2BmCherry in Col-0

Tube: 21-1

Population	#Events	%Parent	%Total
All Events	206,088	####	100.0
P1	194,106	94.2	94.2
P2	186,110	95.9	90.3
P3	36,785	19.8	17.8
P4	18,537	50.4	9.0
neg	14,335	77.3	7.0
P9	9,605	67.0	4.7
P5	159	0.9	0.1

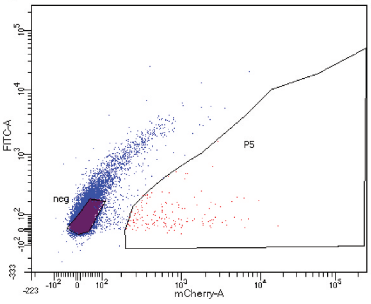

pCLV3::H2BmCherry in *poliv*

Tube: poliv-1

Population	#Events	%Parent	%Total
All Events	200,000	####	100.0
P1	188,169	94.1	94.1
P2	181,988	96.7	91.0
P3	28,667	15.8	14.3
P4	12,949	45.2	6.5
neg	9,029	69.7	4.5
P9	5,207	57.7	2.6
P5	63	0.5	0.0

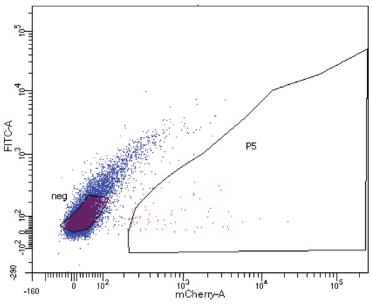

pCLV3::H2BmCherry in *ddm1*

Tube: ddm1-1

Population	#Events	%Parent	%Total
All Events	200,000	####	100.0
P1	188,247	94.1	94.1
P2	186,008	98.8	93.0
P3	17,364	9.3	8.7
P4	8,165	47.0	4.1
neg	4,150	50.8	2.1
P9	2,675	64.5	1.3
P5	355	4.3	0.2

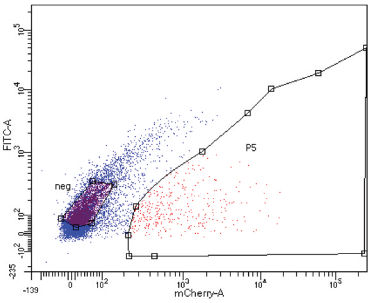

Heat stress

Col-0

Tube: col0

Population	#Events	%Parent	%Total
All Events	400,000	####	100.0
P1	346,113	86.5	86.5
P2	343,661	99.3	85.9
P3	20,917	6.1	5.2
P4	14,936	71.4	3.7
neg	5,802	38.8	1.5
P9	3,199	55.1	0.8
P5	1	0.0	0.0

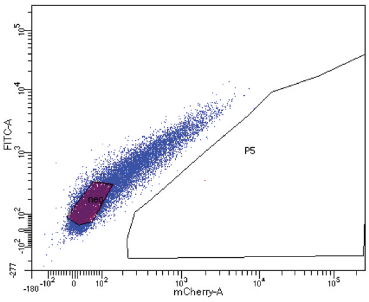

pCLV3::H2BmCherry in Col-0

Tube: 21-1

Population	#Events	%Parent	%Total
All Events	400,000	####	100.0
P1	367,472	91.9	91.9
P2	363,790	99.0	90.9
P3	27,763	7.6	6.9
P4	19,207	69.2	4.8
neg	7,260	37.8	1.8
P9	3,387	46.7	0.8
P5	28	0.1	0.0

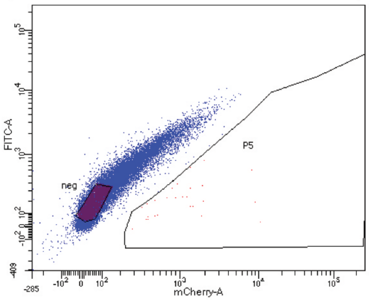

pCLV3::H2BmCherry in *poliv*

Tube: Poliv-1

Population	#Events	%Parent	%Total
All Events	400,000	####	100.0
P1	367,682	91.9	91.9
P2	361,522	98.3	90.4
P3	55,127	15.2	13.8
P4	39,364	71.4	9.8
neg	10,694	27.2	2.7
P9	3,120	29.2	0.8
P5	22	0.1	0.0

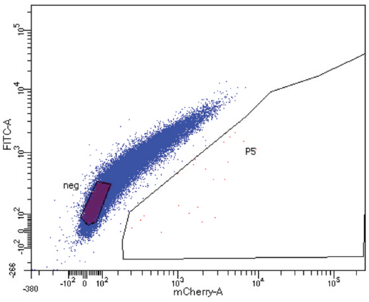

pCLV3::H2BmCherry in *ddm1*

Tube: ddm1-1

Population	#Events	%Parent	%Total
All Events	400,000	####	100.0
P1	377,113	94.3	94.3
P2	371,941	98.6	93.0
P3	67,907	18.3	17.0
P4	52,530	77.4	13.1
neg	10,099	19.2	2.5
P9	2,786	27.6	0.7
P5	79	0.2	0.0

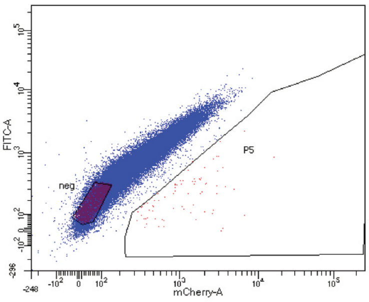

B

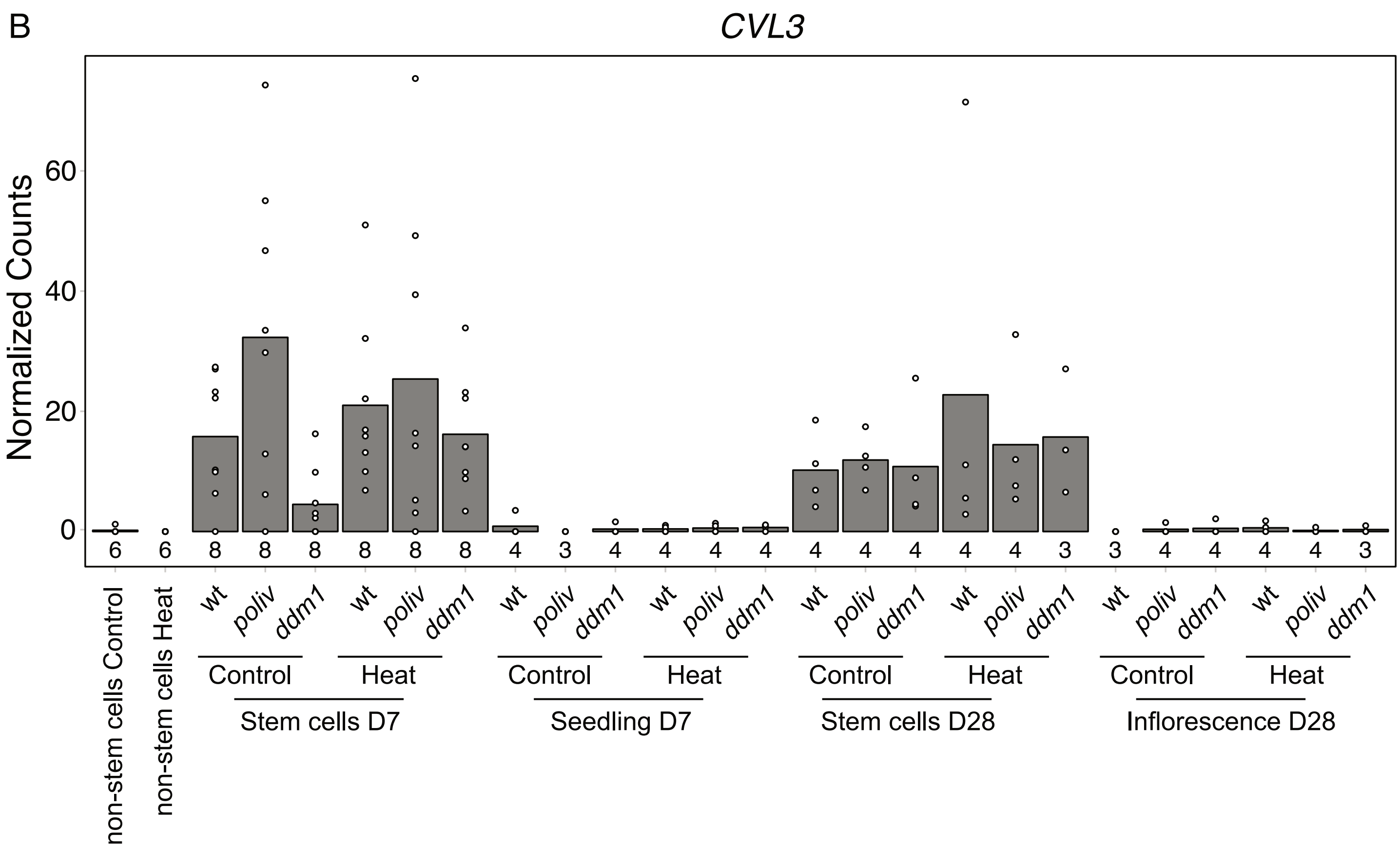

Supplemental Figure S3. Collection of stem cell nuclei marked with fluorescence proteins. (A) Flow cytometry plots showing the results of fluorescence-activated nuclei sorting for control (left) and heat stressed (right) samples of Col-0 and Col-0 wt, *poliv*, and *ddm1* containing the *pCLV3::H3BmCherry* reporter. Gate P5 highlights the number of stem cell nuclei detected per runtime (200,000 or 400,000 events). (B) Normalized *CLV3* counts are shown for all samples. Sample sizes (N) are indicated under the bars.

A

PCA analysis of D7 samples

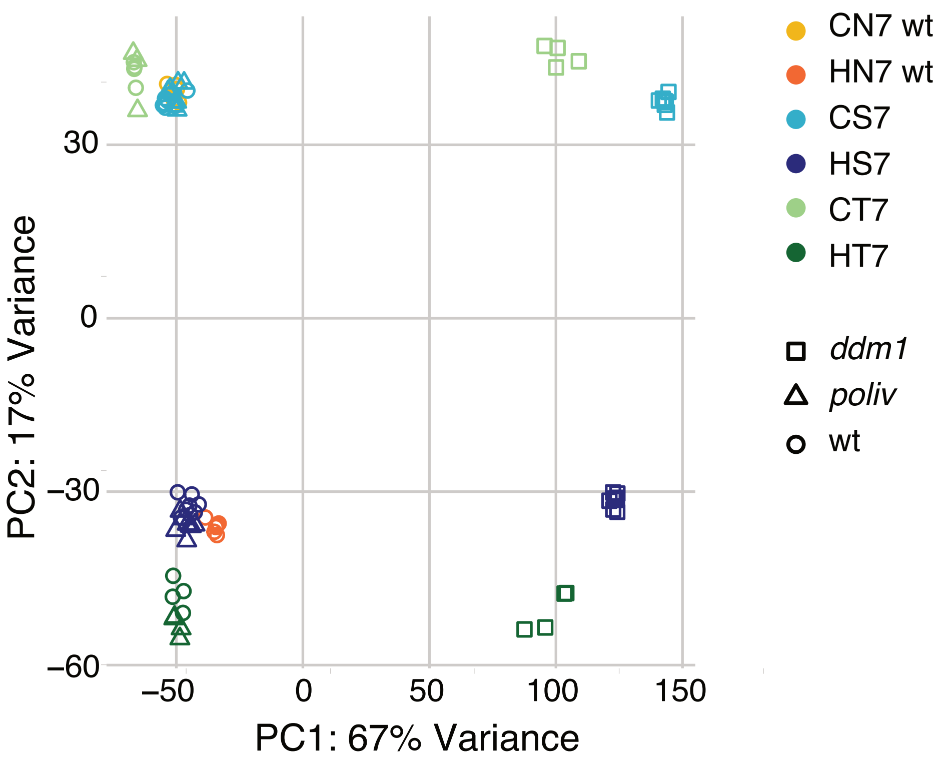

B

PCA analysis of D28 samples

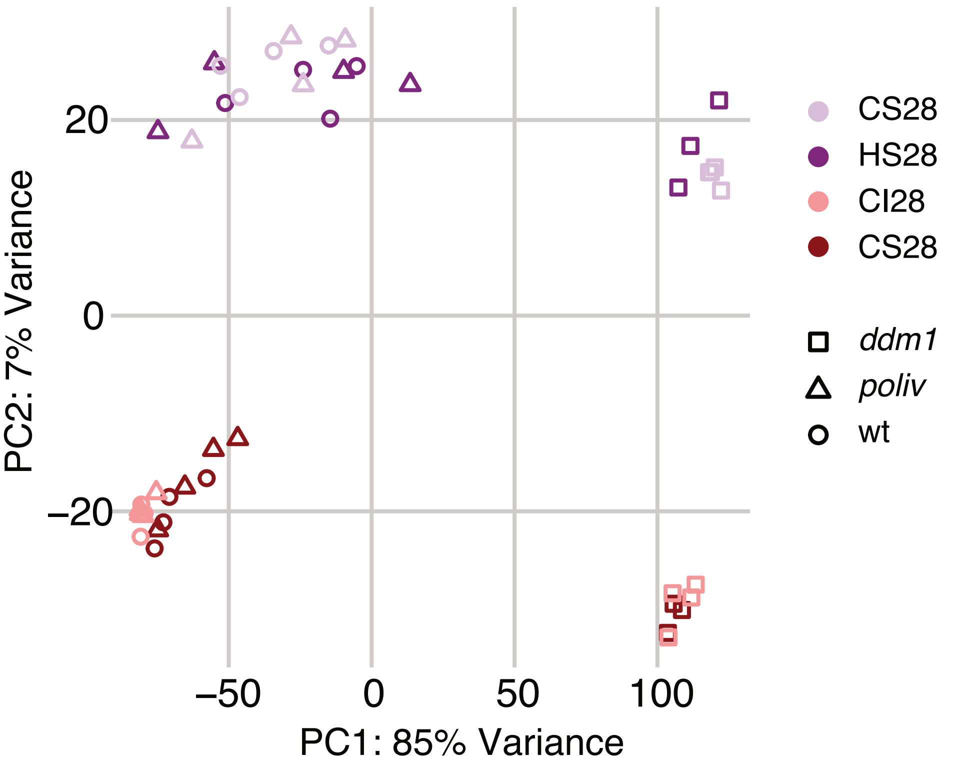

Supplemental Figure S4. Principal component analysis (PCA) of mRNA data, separated by chronological age of the samples. (A) D7 samples collected 7 days after germination. (B) D28 samples collected 28 days after germination. wt, *poliv*, *ddm1* depict genotypes; C: grown at regular temperature; H: grown at elevated temperature; I: Inflorescence: T: whole seedling tissue; S: sorted stem cell nuclei; N: sorted nuclei not expressing the stem cell marker.

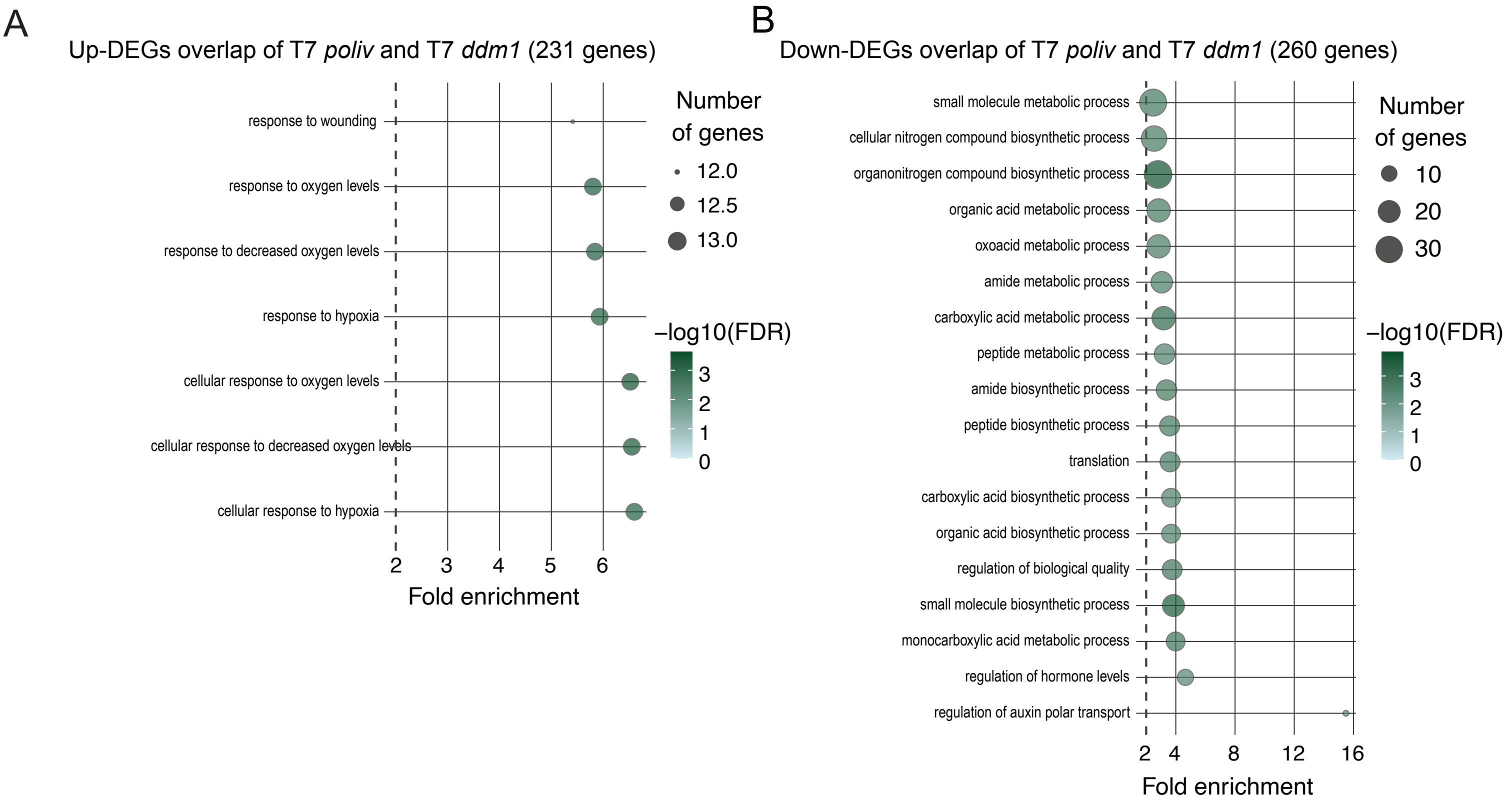

Supplemental Figure S5. Gene ontology analysis of uniquely up- and down-regulated genes in seedlings. (A) A total of 231 up-regulated genes and (B) 260 down-regulated genes are shared between *poliv* and *ddm1*. T7 = seedlings from plants 7 days post-germination.

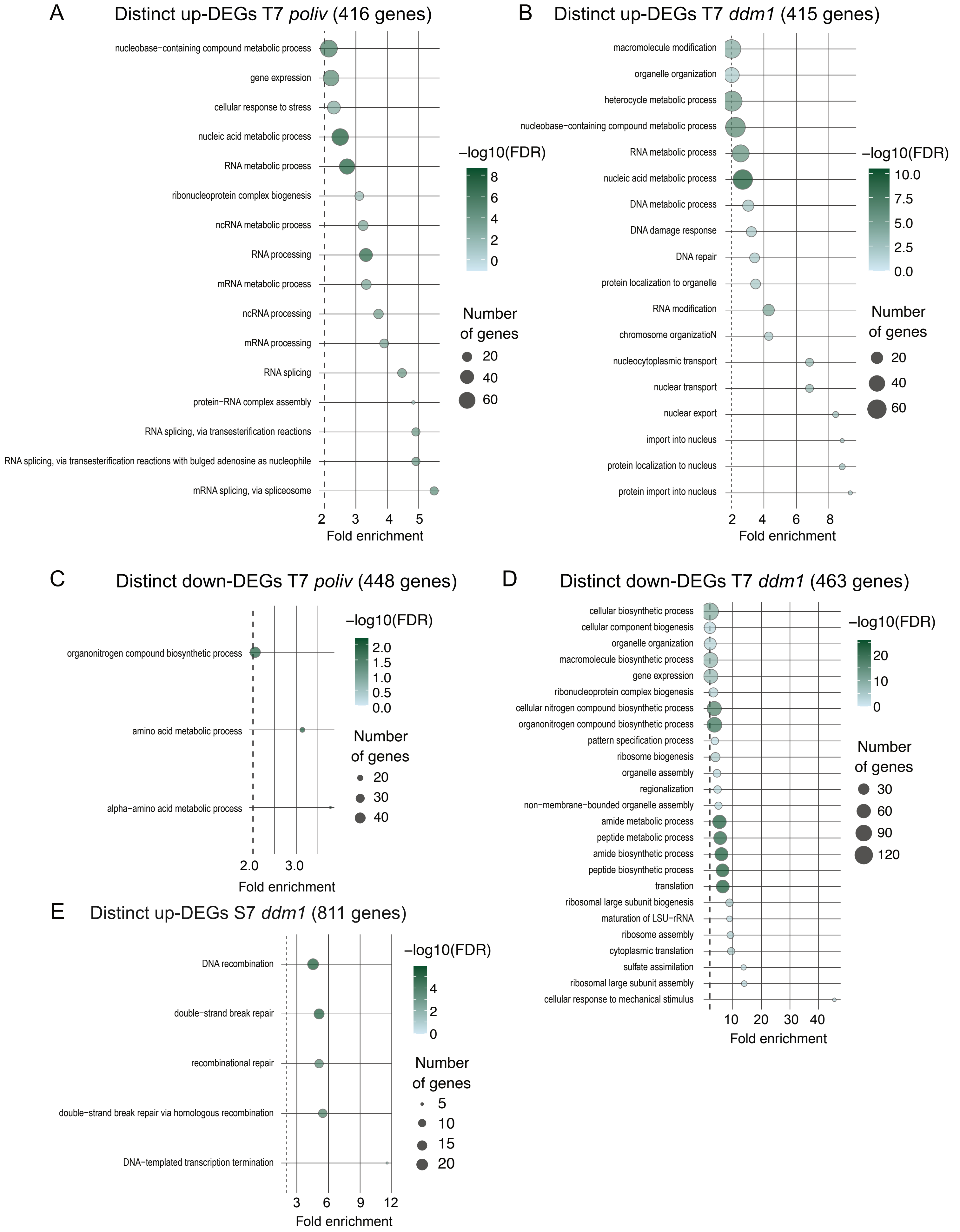

Supplemental Figure S6. Gene ontology analysis of various gene sets. (A) Distinct up-regulated genes in *poliv* and (B) *ddm1* seedling. (C) Distinct down-regulated genes in *poliv* and (D) *ddm1* seedlings. (E) Distinct up-regulated genes in *ddm1* stem cells. T7 = Tissue (seedlings), S7 = stem cell nuclei isolated from plants 7 days post-germination.

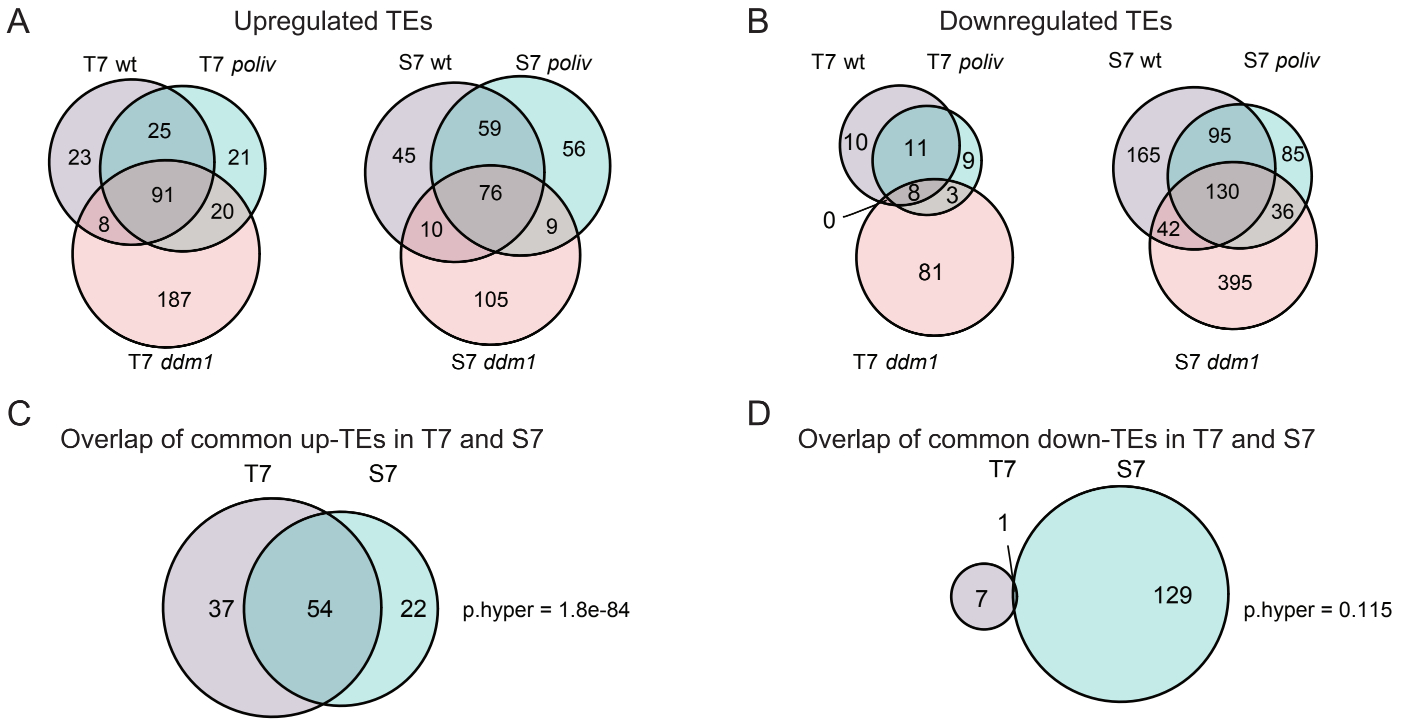

Supplemental Figure S7. Venn diagrams illustrating differentially expressed transposons (TEs) in seedlings (T7) and stem cells at day 7 (S7). **(A)** Up-regulated TEs. **(B)** Down-regulated TEs. **(C)** Common TEs upregulated in seedlings (91) and stem cells (76). **(D)** Common TEs down-regulated in seedlings (8) and stem cells (130).
